# Assessing Progress Toward the Vision of a Comprehensive, Shared Electronic Care Plan: Scoping Review

**DOI:** 10.2196/36569

**Published:** 2022-06-10

**Authors:** Jenna M Norton, Alex Ip, Nicole Ruggiano, Tolulope Abidogun, Djibril Souleymane Camara, Helen Fu, Bat-Zion Hose, Saadia Miran, Chun-Ju Hsiao, Jing Wang, Arlene S Bierman

**Affiliations:** 1 Division of Kidney, Urologic and Hematologic Diseases National Institute of Diabetes and Digestive and Kidney Diseases National Institutes of Health Bethesda, MD United States; 2 School of Medicine and Health Sciences George Washington University Washington, DC United States; 3 School of Social Work, University of Alabama Tuscaloosa, AL United States; 4 Office of Clinical Research Support National Institute of Diabetes and Digestive and Kidney Diseases National Institutes of Health Bethesda, MD United States; 5 Public Health Informatics Fellowship Program Center for Surveillance, Epidemiology, and Laboratory Services Centers for Disease Control and Prevention Atlanta, GA United States; 6 Center for Evidence and Practice Improvement Agency for Healthcare Research and Quality Rockville, MD United States; 7 Richard M Fairbanks School of Public Health Center for Biomedical Informatics, Regenstrief Institute Indiana University Indianapolis, IN United States; 8 Department of Anesthesiology and Critical Care Perelman School of Medicine University of Pennsylvania Philadephia, PA United States; 9 College of Nursing Florida State University Tallahassee, FL United States

**Keywords:** electronic care plan, care planning, care plan, care coordination, multiple conditions, multiple chronic conditions, chronic disease, chronic condition, electronic care, digital health, electronic tools, e-care, healthcare data, eHealth

## Abstract

**Background:**

Care plans are central to effective care delivery for people with multiple chronic conditions. But existing care plans—which typically are difficult to share across care settings and care team members—poorly serve people with multiple chronic conditions, who often receive care from numerous clinicians in multiple care settings. Comprehensive, shared electronic care (e-care) plans are dynamic electronic tools that facilitate care coordination and address the totality of health and social needs across care contexts. They have emerged as a potential way to improve care for individuals with multiple chronic conditions.

**Objective:**

To review the landscape of e-care plans and care plan–related initiatives that could allow the creation of a comprehensive, shared e-care plan and inform a joint initiative by the National Institutes of Health and the Agency for Healthcare Research and Quality to develop e-care planning tools for people with multiple chronic conditions.

**Methods:**

We conducted a scoping review, searching literature from 2015 to June 2020 using Scopus, Clinical Key, and PubMed; we also searched the gray literature. To identify initiatives potentially missing from this search, we interviewed expert informants. Relevant data were then identified and extracted in a structured format for data synthesis and analysis using an expanded typology of care plans adapted to our study context. The extracted data included (1) the perspective of the initiatives; (2) their scope, (3) network, and (4) context; (5) their use of open syntax standards; and (6) their use of open semantic standards.

**Results:**

We identified 7 projects for e-care plans and 3 projects for health care data standards. Each project provided critical infrastructure that could be leveraged to promote the vision of a comprehensive, shared e-care plan. All the e-care plan projects supported both broad goals and specific behaviors; 1 project supported a network of professionals across clinical, community, and home-based networks; 4 projects included social determinants of health. Most projects specified an open syntax standard, but only 3 specified open semantic standards.

**Conclusions:**

A comprehensive, shared, interoperable e-care plan has the potential to greatly improve the coordination of care for individuals with multiple chronic conditions across multiple care settings. The need for such a plan is heightened in the wake of the ongoing COVID-19 pandemic. While none of the existing care plan projects meet all the criteria for an optimal e-care plan, they all provide critical infrastructure that can be leveraged as we advance toward the vision of a comprehensive, shared e-care plan. However, critical gaps must be addressed in order to achieve this vision.

## Introduction

Multiple chronic conditions affect 1 in 3 American adults and 4 in 5 Medicare beneficiaries. It is the most common chronic condition seen in clinical practice, and while there is no standard definition or measure of multiple chronic conditions, it is generally understood to be the co-occurrence of 2 or more chronic mental or physical health conditions. Other impairments or disabilities are also sometimes included in the definition of multiple chronic conditions, as are syndromes such as frailty and social factors such as homelessness. Providing integrated person-centered care to people living with multiple chronic conditions is a major challenge [1,2]. People with multiple chronic conditions and their caregivers often experience significant burdens associated with coordinating care across multiple disease states, clinicians, and settings, including scheduling multiple medical appointments, managing complex drug and dietary regimens, and integrating multiple sources of (sometimes conflicting) medical advice [3-6]. Fragmentation of care for people living with multiple chronic conditions presents multiple challenges to clinicians and contributes to avoidable hospitalizations, duplication of services, adverse events, and higher health care costs [7]. Further, given the disproportionate prevalence of multiple chronic conditions in Black and Hispanic Americans [8], such fragmentation of care may exacerbate disparities in health outcomes.

Care plans are a central component of effective care delivery for people with multiple chronic conditions and other complex health care needs. Care plans, increasingly required by the Center for Medicare and Medicaid Services (CMS) in its programs, include written, comprehensive, patient-centered longitudinal plans of action that identify a patient’s goals and health needs and the services and support required to meet them.

Existing care plans are largely paper based, and when electronic, often designed for a specific care setting or condition. They are often not interoperable and are difficult to share between providers, patients, and caregivers. People with multiple chronic conditions are more likely to have multiple care plans, which, rather than improving care coordination and integration, can instead lead to competing plans and increased fragmentation of care.

A comprehensive, shared electronic care (e-care) plan (CSeCP) that is also interoperable is a dynamic electronic tool that employs health information technology to facilitate collaboration between individuals and their clinical teams, with the goal of addressing the totality of their health and social needs across all care settings [9]. Ideally, a CSeCP would allow clinicians, patients, and caregivers to electronically view role-specific information [10]. A National Quality Forum report on care coordination recommended that an e-care plan should include the following sections, with data shared across all care settings: (1) prioritized health concerns, including social determinants of health (SDoH), (2) health and life goals, (3) interventions, and (4) health status of the individual [11]. Potential benefits of e-care plans include (1) improved quality and efficiency of care, (2) streamlined access to patient health records across the care team (including the patient), (3) coordinated medication and treatment management, and (4) improved care transitions [12-15]. E-care plans can also aid in the assessment, identification, and collection of information on SDoH for individuals and communities and inform practice and policy recommendations across health care settings [16].

The use of CSeCPs has emerged as a potential solution for improving and coordinating the care of individuals with multiple chronic conditions [17]. However, e-care plans that use different data standards cannot be easily shared across providers. Emerging standards combined with commonly used clinical terminology provide a foundation that makes the development of a comprehensive, interoperable e-care plan achievable. The Office of the National Coordinator for Health Information Technology has set a goal of nationwide interoperability by 2024 [17]. This has contributed to a rapid uptake of emerging health information technology data standards, such as the Fast Healthcare Interoperability Resources (FHIR) specification—a flexible standard for exchanging health care information electronically—and Substitutable Medical Applications, Reusable Technologies (SMART), an open, vendor-agnostic, standards-based technology platform that enables the development of applications that seamlessly and securely integrate with health information technology systems [18,19].

To advance toward a CSeCP, the Agency for Healthcare Research and Quality (AHRQ) and the National Institute for Diabetes and Digestive and Kidney Diseases (NIDDK) are collaborating to build interoperable, open-source, patient-, caregiver-, and clinician-facing e-care plan applications and a Health Level Seven (HL7) FHIR implementation guide to improve care coordination for people with multiple chronic conditions across clinical and community settings. To inform this and other efforts in the field, we conducted a scoping review of past and current e-care plans and care plan–related initiatives, aiming to identify foundational projects and resources that could inform the multiple chronic conditions e-care plan project and other efforts in this space. This paper describes the process and results of our scoping review, as well as the functionality of existing e-care plans and the gaps that need to be addressed in order to advance toward a comprehensive e-care plan.

## Methods

Using the scoping review methodology [20], we first searched Scopus, Clinical Key, and PubMed for articles featuring nonproprietary e-care plan projects; the reference list was reviewed to identify additional articles. We also searched the grey literature, used Google, and used others sources such as the US Office of the National Coordinator for Health Information Technology (ONC) Interoperability Proving Grounds [21] and Health Level Seven International [22], an organization accredited by the American National Standards Institute to develop health standards. All searches included combinations of the following terms: “interoperability,” “electronic care plan,” “care plan,” “SMART on FHIR,” “FHIR,” “C-CDA,” and “multiple chronic conditions.” Searches were limited to the years January 2015 to June 2020 to capture recent projects in a rapidly evolving field. In addition, we conducted discussions with expert informants across the federal government, academia, developer and vendor organizations, and industry (including HL7) to identify additional projects missed in the search of gray literature and published literature. Contact information for the included projects was used to identify the informants, who provided individual consultation about e-care plan development. Snowballing techniques [23] were used to add other relevant stakeholders.

Once an e-care plan project was identified, data were extracted, including (1) the implementation period, (2) project contact information, (3) the project description, (4) the population targeted, (5) fields and domains documented through the e-care plan, (6) standard technology features (eg, FHIR and HL7 Consolidated Clinical Document Architecture [C-CDA]), (7) current project activity, and (8) project results and outcomes. To determine how the identified e-care plan, including the multiple chronic conditions e-care plan project, contributes to the development of an interoperable CSeCP, we applied a recently developed typology of care plans by Burt and colleagues [24] that includes three domains: (1) perspective, indicating the degree to which the content and development of the care plan reflect a person- and patient-centered perspective rather than a professional-centered perspective, (2) scope, indicating the focus on discrete behaviors versus broad goals, with an optimal CSeCP including both, and (3) network, or the inclusion of broad care teams rather than patient-clinician dyads. We also expanded on Burt’s typology by adding three domains: (1) context, representing clinical versus SDoH data, with an optimal CSeCP including both, (2) the use of an open syntax (or format) standard (eg, C-CDA or FHIR), and (3) the use of open semantic standards (eg, clinical terminology value sets) to support interoperability. We assessed the degree to which each project met these optimal criteria for a CSeCP.

## Results

### Development of e-Care Plans

**Figure 1:**
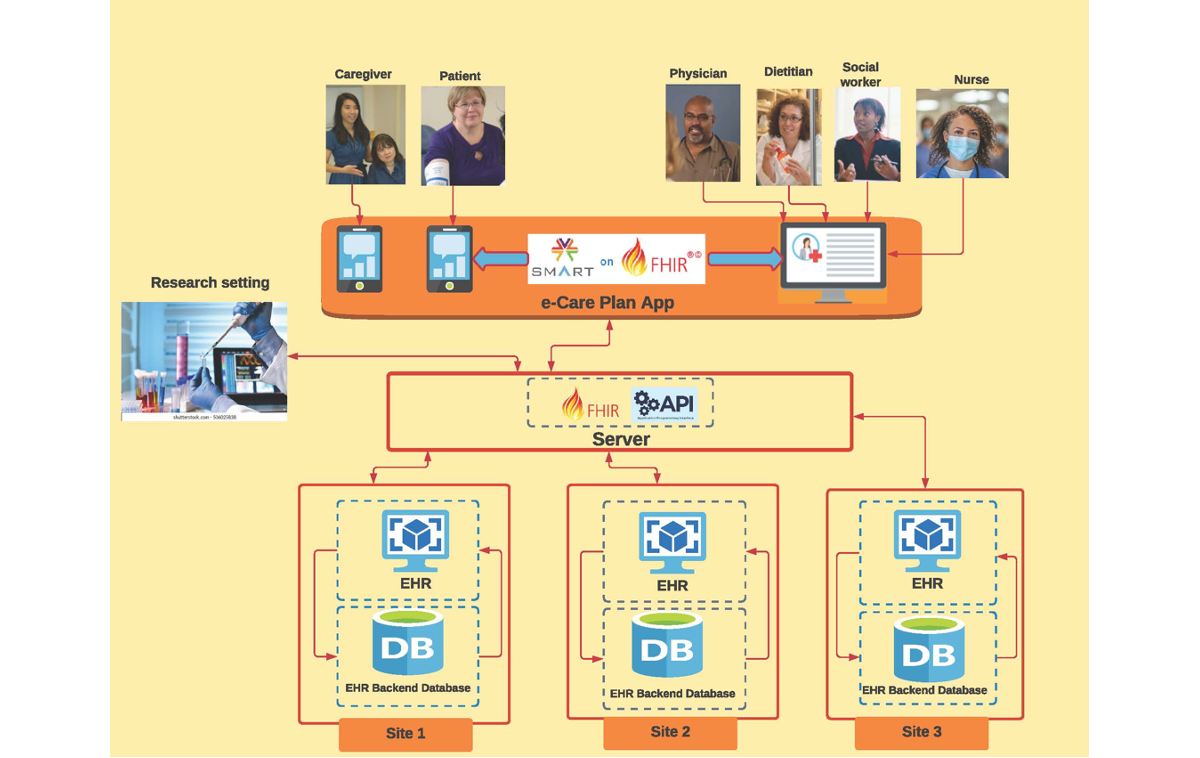
Multiple chronic conditions e-care plan data flow. FHIR: fast healthcare interoperability resources; SMART: substitutable medical applications, reusable technologies; EHR: electronic health records; API: Application Programming Interface.

Table 1 shows the 7 existing nonproprietary e-care plan projects that we identified. These included (1) the Care Plan Domain Analysis Model (DAM) version 1.0, (2) the Care Plan DAM version 2.0 [9,25], (3) the Electronic Long-Term Services and Supports (eLTSS) plan [26], (4) the Pharmacist e-Care Plan (PeCP) [27], (5) the chronic kidney disease (CKD) e-care plan [28], (6) the Dynamic Care Planning (DCP) profile [29], and (7) the Omnibus Care Plan (OCP) [30,31]. Several of these care plans incorporated components of the Standards and Interoperability Framework developed by the National Quality Forum [11] and hence were useful to consider when developing a comprehensive, interoperable e-care plan for multiple chronic conditions. For example, the PeCP initiative includes prioritized health concerns, goals (ie, medication optimization), and interventions (eg, medication management) [27]. Table 1 provides an overview of the e-care plan projects. Figure 1 provides a visual description of the expected data flow for the e-care plan apps. A central FHIR server will aggregate data across multiple settings of care. SMART on FHIR e-care plan apps designed for key users (ie, patients, unpaid caregivers, and clinicians) will pull from the FHIR server to display aggregated patient data. In addition, the apps will collect novel person-centered data and share these data back to the FHIR server, where they will be available (along with comprehensive EHR data) back to clinical and research settings.

**Table 1:** Projects to develop e-care plans.

Organization	Project	Time frame	Description	Users/settings	Domains/features	Underlying standards	Outputs	Contributions to a CSeCP^a^ and gaps
Health Level Seven	Care Plan DAM^b^ 1.0	2011-2016	Provides industry with a set of comprehensive clinical requirement–driven use cases and logical information models to inform design, development, and implementation of care plan systems.	Hospitals; long-term care; home care; mental health	Health concerns (including risks/barriers); goals/preferences; intervention (care activity); outcomes	C-CDA^c^	C-CDA specification	Provides syntax for e-care plans; uses an interdisciplinary approach; allows for multiple, potentially uncoordinated disease/context-specific plans, which is not patient-centered; does not identify semantic standards or specific value sets; does not capture SDoH^d^ data; document-based format limits real-time data updates
Health Level Seven	Care Plan DAM 2.0	2017-present	Uses iterative literature/use case reviews and industry engagement to provide an evidence-based and user-centered blueprint to inform a revision of the Care Plan DAM 1.0 C-CDA specification, develop a FHIR^e^ care plan template, and improve related resources.	Hospitals; long-term care; home care; mental health	DAM 1.0 features plus possible additions: assessment; SDoH; protocol; order/order set (as intervention/care activity); advance directives; care coordination	C-CDA; FHIR	C-CDA specification; FHIR specification	Provides syntax structure for the e-care plan; uses an interdisciplinary approach; allows for multiple, potentially uncoordinated disease/context-specific plans, which is not patient-centered; does not identify semantic standards or specific value sets
Center for Medicare and Medicaid Services and Office of the National Coordinator for Health Information Technology	eLTSS^f^ Initiative	2014-present	Working to identify and harmonize electronic standards to enable the creation, exchange, and reuse of interoperable service plans to improve the coordination of health and social services that support an individual’s mental and physical health.	Long-term service providers (clinical and community); recipients of long-term care	Medicare/Medicaid beneficiary demographics; goals and strengths; person-centered planning; plan information; plan signatures; risks; service information; service provider information	C-CDA; FHIR; clinical terminology	C-CDA implementation guide; FHIR implementation guide; VSAC^g^	Provides semantic standards and value sets for inclusion in a multiple chronic condition e-care plan; provides a syntax for the exchange of data among long-term services and support providers; discipline-specific approach may limit application in the multiple chronic conditions context
Pharmacy Health Information Technology Collaborative	Pharmacist e-Care Plan	2015-present	Provides a standard for interoperable exchange of consensus-driven, prioritized, medication-related activities, plans, and goals for enhanced medication management, specified through Health Level Seven C-CDA and FHIR implementation guides.	Pharmacists; people receiving care in the community; family caregivers; pharmacies; hospitals; long-term care facilities	Patient goals; health concerns; active medication list; drug therapy problems; laboratory results; vitals; payer information; billing for services	C-CDA; C-CDA on FHIR; clinical terminology	C-CDA implementation guide; FHIR implementation guide; VSAC	Provides value sets for inclusion in a multiple chronic conditions e-care plan; provides a syntax for exchange with community-based settings; the discipline-specific approach may limit application in the multiple chronic condition context; document-based format limits real-time data updates
National Institute of Diabetes and Digestive and Kidney Disease	CKD^h^ e-Care Plan	2016-2019	Aimed to facilitate the longitudinal transfer of key patient data among the patient, family caregivers, and the clinical care team across settings by identifying and prioritizing a comprehensive set of clinical and contextual data elements and associated data standards from widely used clinical terminologies.	People with CKD; family caregivers; diverse clinicians providing care for people with CKD; primary care; specialty practices; hospitals	Header (person and plan information); health and social concerns; patient and clinician goals; interventions; health status evaluation and outcomes	Clinical terminology	Value sets specifying more than 300 data elements	Provides value sets for inclusion in a multiple chronic conditions e-care plan; disease-specific approach is of limited use in the context of multiple chronic conditions
Integrating the Healthcare Enterprise	Dynamic Care Planning Profile	2016-present	Provides the structures and transactions for care planning, creating, dynamically updating, and sharing care plans. This profile does not define or assume a single care plan for a patient, but rather depicts how multiple care plans can be shared and used to coordinate care.	Clinicians; patients; payers	Health issues; goals; interventions; outcomes	FHIR; care plan DAM	Supplement to the Integrating the Healthcare Enterprise Patient Care Coordination Technical Framework (Standard for Trial Use 4)	Interdisciplinary approach; allows for multiple, potentially uncoordinated disease/context-specific plans, which is not patient-centered; does not identify specific value sets
SAMHSA^i^	Omnibus Care Plan	2018	Developed SMART^j^ on FHIR, a browser-based (desktop or mobile), patient-centered care coordination application designed to share information with multiple care providers. It is built on existing SMART applications which determine consent, explanation of benefits, and clinical value sets, some of which are proprietary.	Clinicians	Opioid management; suicide prevention; care coordination; alerts/notifications; consent management; task/activity management; referral management; scheduling/ appointments	FHIR; SMART on FHIR	SMART on FHIR application	Provides an open-source SMART on FHIR application for use by clinicians; addresses SDoH and behavioral considerations; use of proprietary tools and applications creates a barrier to implementation and interoperability
Agency for Healthcare Research and Quality, National Institute of Diabetes and Digestive and Kidney Disease, and Assistant Secretary for Planning and Evaluation	Multiple chronic conditions e-care plan	2019-2023	Developing patient- and clinician-facing, interoperable e-care plan applications and a FHIR implementation guide to facilitate aggregation and sharing of critical patient-centered data across home, community, clinic, and research-based settings by extracting data from point-of-care health systems and allowing transfer of that data across settings.	People with multiple chronic conditions, including CKD, type 2 diabetes, cardiovascular disease, and chronic pain; family caregivers; diverse clinicians providing care for people with multiple chronic conditions; home and community-based providers	Person/plan information; health and social concerns; patient and clinician goals; interventions; health status evaluation and outcomes	FHIR; SMART on FHIR; clinical terminology	FHIR implementation guide; clinician SMART on FHIR app; patient mobile SMART on FHIR app	Provides syntax and semantic standards for the exchange of patient data across multiple users/settings; provides a proof-of-concept of a single comprehensive shared care plan; will require expansion to additional disease states

^a^CSeCP: comprehensive shared electronic (e-)care plan

^b^DAM: domain analysis model

^c^C-CDA: consolidated clinical document architecture

^d^SDoH: social determinants of health

^e^FHIR: Fast Healthcare Interoperability Resources

^f^eLTSS: electronic long-term services and supports

^g^VSAC: Value Set Authority Center [32]

^h^CKD: chronic kidney disease

^i^SAMHSA: Substance Abuse and Mental Health Services Administration

^j^SMART: substitutable medical applications, reusable technologies

**Figure 2:**
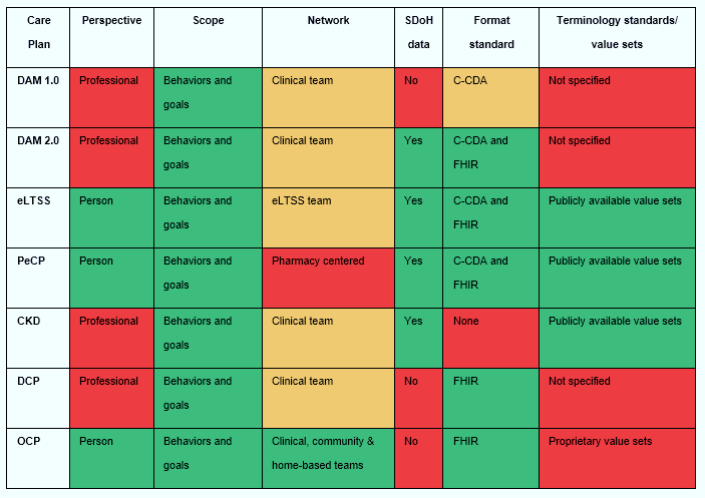
Alignment of identified care plan projects with comprehensive, shared electronic care plan criteria. Red indicates suboptimal alignment with a criterion, yellow indicates partial alignment, and green indicates optimal alignment. DAM: domain analysis model; C-CDA: consolidated clinical document architecture; CKD: chronic kidney disease; eLTSS: electronic long-term services and supports; FHIR: fast healthcare interoperability resources; SDoH: social determinants of health; SMART: substitutable medical applications, reusable technologies; PeCP: pharmacist e-care plan; DCP: dynamic care planning; OCP: omnibus care plan; CSeCP: comprehensive, shared electronic care plan.

Figure 2 shows a schema of the degree to which each project aligned with the optimal CSeCP criteria. We determined that—in the context of multiple chronic conditions—4 of the 7 care plan projects reflected the perspective of the professional rather than that of the person, because they either supported only a single disease (eg, a CKD e-care plan), or they allowed for multiple, distinct and potentially uncoordinated disease or context-specific plans (DAM 1.0, DAM 2.0, and DCP). Either situation—a single disease care plan or multiple uncoordinated care plans—would not meet the needs of a person with multiple chronic conditions who must manage their conditions simultaneously in their day-to-day life, and thus does not reflect such a person’s perspective. All plans supported both broad goals and specific behaviors. Only the OCP supported a network of professionals across the clinical, community, and home-based networks, while 4 projects supported the entire clinical team, 1 supported the entire LTSS team, and 1 focused primarily on pharmacist care. Four of the care plan projects (DAM 2.0, eLTSS, PeCP, and CKD) included SDoH data. All but the CKD care plan specified either C-CDA, FHIR, or both as syntax standards, while only 3 projects (eLTSS, PeCP, and CKD) specified open-source clinical terminology value sets (eg, Logical Observation Identifiers, Names, and Codes [LOINC], Systematized Nomenclature of Medicine Clinical Terms [SNOMED-CT], International Classification of Diseases 10th Revision [ICD-10], Current Procedural Terminology [CPT], or RxNORM). For instance, the CKD e-care plan project identified data standards from common clinical terminology for more than 300 prioritized data elements, and partnered with the Regenstrief Institute to develop new LOINC codes for the data elements lacking existing data standards [28]. The OCP uses proprietary tools to identify specific condition value sets, creating a barrier for potential implementation and interoperability [30].

### Development of Key Health Care Data Standards for People With Multiple Chronic Conditions

Table 2 shows the 3 projects we identified that are developing clinical terminology and coding harmonization that can be leveraged in the development of interoperable e-care plans. These projects included (1) the Data Element Library (DEL) [33], (2) the Gravity Project [34], and (3) the Post-Acute Care Interoperability (PACIO) project [31]. The DEL specifies data elements and standards for the data that the CMS requires postacute care facilities to collect as part of patient health assessments. The Gravity Project, led by the Social Interventions Research and Evaluation Network at the University of California, San Francisco, is a national collaborative to harmonize documentation of SDoH data in electronic health record (EHR) systems. The PACIO project aims to identify data standards to advance interoperable health data exchange between postacute care providers, other health care providers, patients, and key stakeholders through a consensus-based, case-driven approach. Their initial efforts have focused on data standards relating to cognitive and functional status.

**Table 2:** Development of key health care data standards for people with multiple chronic conditions.

Organization	Project	Time frame	Description	Intended users	Fields/domains	Standards	Outputs
Center for Medicare and Medicaid Services	Data Element Library	2018-present	Centralized resource for Center for Medicare and Medicaid Services assessment instrument data elements (eg, questions and responses) and their associated health information technology standards.	Inpatient rehabilitation facilities, home health agencies, long-term care hospitals, skilled nursing facilities, hospice care, home and community-based services	IRF^a^ Patient Assessment Instrument, Outcome and Assessment Information Set; LTCH^b^ Continuity Assessment Record and Evaluation Data Set; SNF^c^ Minimum Data Set; Hospice Item Set; Functional Assessment Standardized Items	Clinical terminology	Standardized data elements relevant to postacute care
Social Interventions Research and Evaluation Network	Gravity Project	2019-present	Develop structured data standards to reduce barriers to documentation and exchange of social determinants of health data, including social risks and protective factors	Health care	Food insecurity, housing instability and homelessness, inadequate housing, transportation access; additional domains to be determined	FHIR^d^; clinical terminology	Social determinants of health FHIR implementation guide
Center for Medicare and Medicaid Services and The Alliance to Modernize Healthcare	Post-Acute Care Interoperability Project	2019-present	Advance interoperable health data exchange between postacute care and other providers, patients, and key stakeholders across health care.	Postacute care, long-term care hospitals, home health agencies, skilled nursing facilities, inpatient rehabilitation facilities	Cognitive status; functional status; additional domains to be determined	FHIR; clinical terminology	Cognitive status, FHIR implementation guide, functional status FHIR implementation guide

^a^IRF: Inpatient Rehabilitation Facility

^b^LTCH: Long-Term Care Hospital

^c^SNF: Skilled Nursing Facility

^d^FHIR: Fast Healthcare Interoperability Resources

## Discussion

Most care plans in use today are paper-based and localized or limited to a specific discipline, disease, or care setting. An electronic, interoperable CSeCP has the potential to greatly improve the quality of care for individuals with multiple chronic conditions, who see numerous providers across multiple care settings, and overcome barriers faced by these providers to accessing and sharing person-centered health information across settings. The burden of multiple chronic conditions is increasing in the United States as its population ages, warranting a redoubled focus on care coordination and the interoperable exchange of health information for people with multiple chronic conditions. Greater interoperability across all health care settings may improve health outcomes, increase clinician workflow efficiency, decrease redundant services, minimize searching for clinical information, and reduce health care costs. This need is heightened in the wake of the ongoing COVID-19 pandemic, which has increased the use of virtual care and, given evidence of potential long-term complications among COVID 19 survivors, may result in individuals with underlying chronic conditions carrying a heavy burden of multiple chronic conditions, in addition to creating a new cohort of people with multiple chronic conditions in previously healthy populations.

Prior efforts to develop e-care plans [9,14,25,27,29,35,36] and data standards [31,33,34] have provided a solid foundation that makes the realization of a CSeCP more feasible. While none of the existing care plan projects identified by our review met all our criteria for an optimal CSeCP, each provides critical infrastructure that can be leveraged as we advance toward the vision of the CSeCP. The multiple chronic conditions e-care plan project [37] aims to build on the identified e-care plan and standards efforts to bring us closer to a CSeCP. The multiple chronic conditions e-care plan project will support the aggregation and sharing of person-centered data through identification of key data elements and clinical terminology standards, specification of an HL7 FHIR implementation guide, and development of clinician-, patient-, and caregiver-facing SMART on FHIR e-care plan applications. The multiple chronic conditions e-care plan project takes a person-centered approach, aggregating person-important health and social data—including patient-reported outcomes—across numerous chronic conditions, beginning initially with CKD, a subset of cardiovascular diseases (ischemic heart disease, hypertension, and heart failure), type 2 diabetes, and chronic pain. With these conditions as a use case, the project will provide an extensible framework for a CSeCP upon which additional disease- and condition-specific value sets and FHIR profiles can be added. To curate a holistic set of data elements for exchange, data element selection and prioritization are informed by broad stakeholder input through technical expert panels. These technical expert panels consist of people with multiple chronic conditions, their caregivers, clinicians from diverse disciplines, community organizations, clinical informaticists, EHR vendors, and developers, among others. The project focuses not only on the core patient–primary care provider dyad but also on a wide, multidisciplinary care team network across the clinical, community, and home-based settings of care. The draft implantation guide and multiple chronic conditions e-care plan project app is being tested during multiple HL7 Connectathons and implemented and tested across real-world clinical and community-based settings of care, with the goal of balloting through HL7 as a standard for trial use in September 2022.

While we anticipate that the multiple chronic conditions e-care plan project will bring us closer to the vision of a CSeCP, much work will be necessary beyond the scope of this project. Key data elements and corresponding value sets and FHIR profiles must be identified and specified for numerous additional chronic conditions. Many data elements known to be important for care—including SDoH—are currently not supported by semantic standards or clinical terminology. While efforts to build these standards are underway [34], widespread implementation may take years. “Writing back” consolidated care plan data to individual EHRs will be necessary to achieve the full interoperability benefits of the e-care plan; however, writing back remains a widely recognized policy challenge, as many EHR systems are reluctant to write back data from external systems. While standard practices are in place for patient authorization of data exchange on a broad scale, additional work is needed to determine whether and how individuals may wish to specify data access privileges on the individual data element level—and to determine the implications this may have for individual privacy and care coordination. Such data element–level specification may be particularly important for potentially stigmatizing information (eg, sexually transmitted diseases, mental health conditions, or addiction). In addition to the semantic and syntax standards included in this scoping review, the realization of a CSeCP will require a comprehensive reference architecture outlining the structures and integrations of the various information technology products and systems, such as EHRs and health information exchanges, potentially involved in the exchange of e-care plan data. The Centers for Disease Control and Prevention’s Making EHR Data More Available for Research and Public Health (MedMorph) project [38] aims to develop a reliable, scalable, and interoperable reference architecture and demonstrated implementation to access and share EHR data across multiple public health and research scenarios. However, many home- and community-based providers have information technology systems that are distinct from the traditional health information technology infrastructure and do not have health information exchange access. Further, unaffiliated EHRs are more widely used in rural settings, creating a barrier to implementation of e-care plans in areas that are already disproportionately affected by poor health outcomes [39]. Additional work will be necessary to ensure equitable application of the CSeCP and other health information technology solutions regardless of location.

The purposes of an interoperable shared e-care plan are, first, to improve the quality and outcomes of care delivery by improving communication, coordination, and information sharing across clinical teams, patients, and caregivers. The second purpose is to provide comprehensive data on clinical conditions and management, as well as patient-reported outcomes, social factors, and patient goals and preferences in order to conduct real world research on people living with multiple chronic conditions. Clinical research on the management of different constellations of disease and health service research on the most effective models of care delivery are both needed [1]. Furthermore, since common risk factors such as smoking, physical inactivity, and unhealthy diets lead to multiple conditions, research on reducing the risk of developing multiple chronic conditions is also needed. Our study has several strengths. This is the first review to identify and assess the numerous ongoing activities in the dynamic field of care plan development. Our data collection and search strategies were broad. In addition to searching academic literature, we reviewed the gray literature, including the use of search engines and a review of government and standards development organization websites, and conducted stakeholder interviews. However, we must acknowledge certain limitations. Information gathered from websites may not be frequently updated, which could have limited our understanding of specific aspects of the sampled e-care plans and related projects. However, this may have been mitigated by our strategy of interviewing stakeholders. Our focus was limited to nonproprietary plans and e-care plan–related initiatives that have developed data standards to support interoperability. Several identified care plan and standards projects are ongoing, and thus their final outputs and success remain to be seen.

A CSeCP has the potential to greatly improve the quality of care for individuals with multiple chronic conditions who see multiple providers across multiple care settings. Prior efforts to develop e-care plans [9,14,25,27,29,35,36] and data standards [31,33,34] provide a solid foundation that makes the realization of a CSeCP feasible. The multiple chronic conditions e-Care Plan is building on these efforts to advance toward a CSeCP. However, critical gaps must be addressed in order to achieve a person-centered, interdisciplinary, and interoperable CSeCP.

## Abbreviations

AHRQ: Agency for Healthcare Research and Quality
C-CDA: Consolidated Clinical Document Architecture
CKD: chronic kidney disease
CMS: Center for Medicare and Medicaid Services
CSeCP: comprehensive shared electronic (e-)care plan
DAM: Domain Analysis Model
DCP: Dynamic Care Planning
DEL: Data Element Library
EHR: electronic health record
eLTSS: Electronic Long-Term Services and Supports
FHIR: Fast Healthcare Interoperability Resources
HL7: Health Level Seven
NIDDK: National Institute for Diabetes and Digestive and Kidney Diseases
OCP: Omnibus Care Plan
ONC: US Office of the National Coordinator for Health Information Technology
PACIO: Post-Acute Care Interoperability
PeCP: Pharmacist e-Care Plan
SDoH: social determinants of health
SMART: Substitutable Medical Applications, Reusable Technologies
